# Supplementary material for: Patterns of Intron Gain and Loss in Fungi
Source: PLoS Biol. 2004 Nov 30;2(12):e422. doi: 10.1371/journal.pbio.0020422 (PMC532390; doi:10.1371/journal.pbio.0020422)
Supplement: Table S1 — Also available at http://genes.mit.edu/NielsenEtAl/. (4.3 MB ZIP). [file pbio.0020422.st001.zip › NielsenEtAl/html/704.html]

AN2327.1.NCU00736.1.MG05569.1.FG05028.1


```
 CLUSTAL W (1.82) Multiple Sequence Alignments - Introns Inserted


Sequence 1: MG05569.1	490 aa
Sequence 2: FG05028.1	450 aa
Sequence 3: AN2327.1	560 aa
Sequence 4: NCU00736.1	486 aa
Alignment Length: 601 aa
Number Identitical Residues: 157 aa
Alignment Score (without introns) 8605


MG05569.1 	------------------------------------------------------------
NCU00736.1	------------------------------------------------------------
FG05028.1 	------------------------------------------------------------
AN2327.1  	MKTLQYLLKYSLACYLMVIPISSTVSSLLVRSLMWSSEKGLVDFARRAVETPGGDILHVP
          	 .: .   . : :.     . ::: ::    :   ::...  . :  : .:....    .

MG05569.1 	------------------MEEADKRAEELRAQIAECEATLQSLKEQLAAAEAAKTPPYSD
NCU00736.1	--------------------------------MQNGGASSDQNGSISSVPRDTSR-----
FG05028.1 	------------------METVDK----LRSEIEKREVELADLRSQLAAAESAAR-----
AN2327.1  	HAGFLRPTARSYRPNIFWMEDLETRCASLRTEIAAAEAQLTKLKRELHEAEGAALRAQSQ
          	 :.   .:: :  ..    .  .. . .  :.:    .   .       .. :   . :.

MG05569.1 	STETDRGSS--SSTWKWPLAEAEYERYGRQLILPSVGIQG1QLRLKAASVLIVGAGGLGC
NCU00736.1	-----------------PLTEDELDRYSRQMIVPGMGKEA1QLRLINAKVLIIGAGGLGC
FG05028.1 	ETET-------KESWKWPLDNHEYERYSRQMIVPNFGLQG1QLRLRKARVLLVGAGGLGC
AN2327.1  	KTASANATTGQRTKSKWPLHGEEYRRYGRQMIVPQFGLQG1QLKLRDAKVLIVGAGGLGC
          	.: :  .::..  . . **   *  **.**:*:* .* :. **:*  * **::*******

MG05569.1 	PASAYFAGAGVGTIGLVDGDTVEASNLHRQVAHGTSRVGMLKVDSAISYLRE~LNPLVKY
NCU00736.1	PAAQYIAGAGIGTIGIADGDTVERSNLHRQVGHSTSRIGQSKVSSLITHLRG~LNPLPTY
FG05028.1 	PAAAYLAGSGVGTIGLVDGDEVEVSNLHRQIAHSTGRVGMSKVQSAITYLKE2LNPTITY
AN2327.1  	PAALYLAGAGVGTIGLVDGDTVEASNLHRQVLHRSRNVGKLKVDSAIEYLRE2LNPHPTY
          	**: *:**:*:****:.*** ** ******: * : .:*  **.* * :*:  ***  .*

MG05569.1 	NAHQSHLTPENAESIVSGYDLVLDCTDHPTSRYLISDVCVLLRKPLVSASALRTDGQLIV
NCU00736.1	VAHTTHITPLNAADLISQYDLILDCTDNPATRYLISDVCVLLCKPLVSAASVQTSGQIIV
FG05028.1 	NAYNTHLTPQNAQDIVSEYDLVLDCTDHPTSRYLISDICVLLSKPLVSASAFQTSGQLII
AN2327.1  	IAHQAHLTPREAPDIFKDYDLILDCTDNPATRYLISDTAVLLGKPLVSASALRTEGQLMV
          	 *: :*:** :* .:.. ***:*****:*::****** .*** ******::.:*.**:::

MG05569.1 	LNTPAAPQADLSGG---PCYRCVFPKPPPPDAVTSCGEGGILGPVVGVMGVLQALEGIRL
NCU00736.1	LNCPPTPQGQLDGGPYPPCYRCCFKKPPPANAQLSCGEAGILGPVVGLMGVAQAGEAIKI
FG05028.1 	LNNPPG------KG---PCYRCVFPTPPPPDSVVGCGEGGIIGPIVGTMGVLQALETIKL
AN2327.1  	LNNPPQPPGDKTGG---PCYRCVFPKPPPANSVTSCADGGILGPVVGTMGVLQASEAIKV
          	** *. . ..   *   ***** * .***.::  .*.:.**:**:** *** ** * *::

MG05569.1 	LAAGRHL-----------SPSPEQQQTAISPSLLLFSAPPDGSPA--GFRSVRMRGRRKD
NCU00736.1	LASALHIPTTTTTTTSPPAQGVPRAAPVVEPTLLLYSYSLTSFLSPFTFRALKMAPRKKN
FG05028.1 	ILRG----------------DLEIHGEAKTPMLLLFTGTADTP-----FRSVRMRGKRKT
AN2327.1  	LTSAG------------------ESVEATPPSLLIFSAYSSPQ-----FRTIKLRSRRPN
          	:  .                       .  * **:::           **::::  ::  

MG05569.1 	CFACGE--KSALSLATLREGGLDYVQFCGG--SRKPVALLKSEERVSAEQLNALLQ--QQ
NCU00736.1	CFACGEGSKQRLTLEGVKNGEPNYDFFCGLGGGGPEKGVLSEEERITPREFVERVQGKDG
FG05028.1 	CLACGD--EKGLTLEELKTS-MDYVQFCG---VRRSVQLLKPDERVTPKEYEMISK--SG
AN2327.1  	CAVCSA--EATVTLESVRSGSMDYVFFCG---TVDPADILSPEERISPSEYGNVDS--AG
          	* .*.   :  ::*  :: .  :*  ***         :*. :**::. :     .    

MG05569.1 	AGEHGKPVLLDVREREHFEIANIPGAINIPFSKTQNPG--ARHNAEDTPKLD--WLPDGV
NCU00736.1	NGKGKKYVVLDTREKEHFSFGSIEGAVNLPFGKLLSKAAQLKR-SGETPKVGDILPPEIQ
FG05028.1 	DKET---LLVDVREKEHFDLCNISGSINIPISRFMS----ARG--ETTPEG---WPSD--
AN2327.1  	AQRH----IIDVREKVQFDICSLENSINIPMSTILASAYSAPTLDADEPKRLPSWLPPEV
          	  .     ::*.**: :*.: .: .::*:*:.     . .        *:      .   

MG05569.1 	ADGHS-----PVYVVCRVGNDSQTVARQLKEFGLDNQGKRFIGDVKGGMLAWKREVDSTL
NCU00736.1	VRDGHGDKDIPIYVVCRRGLDSQEAVEKLKEMGLDNGGSRKIVDIAGGMKAWKEQVDPSF
FG05028.1 	LSPST-----PIYLVCRVGNDSQIAAQKLKTLGIGKNGERFIGDISGGIKSWKDTVDPTV
AN2327.1  	AHESNK----PIYVVCRQGNDSQTVVRKLKELGLDHGGERPVVDIKGGFRSWREQVDPDW
          	          *:*:*** * *** ...:** :*:.: *.* : *: **: :*:  **.  

MG05569.1 	PFM
NCU00736.1	PYL
FG05028.1 	PFI
AN2327.1  	PDY
          	*
```
